# Polyphenols from *Lycium barbarum* (Goji) Fruit European Cultivars at Different Maturation Steps: Extraction, HPLC-DAD Analyses, and Biological Evaluation

**DOI:** 10.3390/antiox8110562

**Published:** 2019-11-16

**Authors:** Andrei Mocan, Francesco Cairone, Marcello Locatelli, Francesco Cacciagrano, Simone Carradori, Dan C. Vodnar, Gianina Crișan, Giovanna Simonetti, Stefania Cesa

**Affiliations:** 1 Department of Pharmaceutical Botany, “Iuliu Haţieganu” University of Medicine and Pharmacy, 23 Gheorghe Marinescu Street, 400337 Cluj-Napoca, Romania; mocan.andrei@umfcluj.ro (A.M.); gcrisan@umfcluj.ro (G.C.); 2 Laboratory of Chromatography, Institute of Advanced Horticulture Research of Transylvania, University of Agricultural Sciences and Veterinary Medicine, 400372 Cluj-Napoca, Romania; 3 Dipartimento di Chimica e Tecnologie del Farmaco, Università degli Studi di Roma “La Sapienza”, P.le Aldo Moro 5, 00185 Rome, Italy; francesco.cairone@uniroma1.it; 4 Department of Pharmacy, “G. d’Annunzio” University of Chieti–Pescara, Via dei Vestini 31, 66100 Chieti, Italy; marcello.locatelli@unich.it (M.L.); francesco.cacciagrano@studenti.unich.it (F.C.); simone.carradori@unich.it (S.C.); 5 Department of Food Science, University of Agricultural Sciences and Veterinary Medicine, 400372 Cluj-Napoca, Romania; dan.vodnar@usamvcluj.ro; 6 Dipartimento di Sanità Pubblica e Malattie Infettive, Università degli Studi di Roma “La Sapienza” P.le Aldo Moro 5, 00185 Rome, Italy; giovanna.simonetti@uniroma1.it

**Keywords:** goji berry, *Lycium barbarum*, HPLC-DAD, antioxidant capacity, TPC, TFC, anti-tyrosinase activity, anti-*Candida* activity

## Abstract

Goji berries are undoubtedly a source of potentially bioactive compounds but their phytochemical profile can vary depending on their geographical origin, cultivar, and/or industrial processing. A rapid and cheap extraction of the polyphenolic fraction from *Lycium barbarum* cultivars, applied after homogenization treatments, was combined with high-performance liquid chromatography (HPLC) analyses based on two different methods. The obtained hydroalcoholic extracts, containing interesting secondary metabolites (gallic acid, chlorogenic acid, catechin, sinapinic acid, rutin, and carvacrol), were also submitted to a wide biological screening. The total phenolic and flavonoid contents, the antioxidant capacity using three antioxidant assays, tyrosinase inhibition, and anti-*Candida* activity were evaluated in order to correlate the impact of the homogenization treatment, geographical origin, and cultivar type on the polyphenolic and flavonoid amount, and consequently the bioactivity. The rutin amount, considered as a quality marker for goji berries according to European Pharmacopeia, varied from ≈200 to ≈400 µg/g among the tested samples, showing important differences observed in relation to the influence of the evaluated parameters.

## 1. Introduction

*Lycium barbarum* L. is a traditional Chinese medicinal plant, specifically a shrub belonging to Solanaceae family, whose fruits, well known as goji berries, have acquired increasing popularity in Europe and North America in recent years. Goji berries and their derived products are considered a relevant source of (micro)nutrients, especially natural antioxidants, which contribute to the extraordinary nutritional quality of this matrix [1]. In fact, a healthy functional role is recognized as belonging to its fruits and their derived extracts and infusions, containing polysaccharides, carotenoids, and flavonoids, as well as salts, vitamins, and other micronutrients. The original habitat of goji is not certainly established, although more than 70 different species of *Lycium* exist [2]; *L. barbarum* represents the most relevant species from a biological point of view [3,4]. Overall, the chemical diversity of this fruit could provide many opportunities for food supplement development given the important growth forecasts of the polyphenol market worldwide.

Actually, the most researched components from goji fruits are the water-soluble arabinogalactans (*Lycium barbarum* polysaccharides or LBP), which are estimated to comprise up to 5–8% of the dried fruits [5] and include in their composition six types of monosaccharides (arabinose, rhamnose, xylose, mannose, galactose, and glucose), galacturonic acid, and almost all the proteinogenic amino acids [6]. LBP have been shown to control blood glucose, modulate glucose metabolism leading to the improvement of oxidative stress markers, alleviate insulin resistance, and stimulate the increase of glucose transporter type 4 (GLUT4) expression [7,8].

Dried fruits are composed of a carotenoid fraction of 0.03–0.5% [9], in which 11 free carotenoids and 7 carotenoid esters were detected from unsaponified and saponified *L. barbarum* extracts [6]. Zeaxanthin dipalmitate, which can vary from 30% to 80% of total carotenoids, represents the main molecule [10], followed by β-carotene, neoxanthin, and cryptoxanthin at lower concentrations. The carotenoid content of goji berry was recently the object of great interest for researchers for its beneficial effects on retinopathy. The ability to protect the visual function and the overall antioxidant properties [11] make this matrix an interesting ally for the prevention of the onset and the care of age-related macular degeneration (AMD), which represents one of the main causes of vision loss, which is estimated as cause of 5% of all cases of blindness [12]. Among carotenoids, lutein and zeaxanthin, the most represented pigments in the macular area of the human retina, are particularly effective in the macula protection from oxidative damage by scavenging harmful reactive oxygen species. Therefore, lutein and zeaxanthin may play a pivotal role in preventing AMD if further studies can clarify the explicit effects that can be expected in terms of their function regarding the development and progression of AMD [13].

Concerning phenylpropanoids, flavonoids, and isoflavonoids, the goji berry’s protective and antioxidant role was correlated with the presence of caffeic and chlorogenic acid, with a high content of quercetin-3-*O*-rutinoside and kaempferol-3-*O*-rutinoside, and finally with representative coumarins and lignans [14,15]. Furthermore, phenolics are the most abundant secondary metabolites of plants [16]. Quercetin, kaempferol, and relative derivatives, among which rutin is the most frequent and representative, are well-known as radical scavengers and anti-oxidants capable of preventing cancer, cardiovascular disease, and other chronic disease onset [3,17]. In recent years, polyphenols were shown to exert a protective role against neurodegeneration, an ability related to their antioxidant and scavenging abilities. They could interact positively with vitamins E and C in lipid bilayer membranes via non-covalent associations, enabling the regeneration of these endogenous antioxidants [18,19].

Very recently, goji extracts have also been considered as potential candidates for designing innovative functional products (cosmetics and cosmeceuticals, among others) from natural matrices in the treatment of pigmentation disorders, and *L. barbarum* has also shown the capacity to inhibit the oxidation of L-DOPA, which is catalyzed by tyrosinase [20,21,22]. Tyrosinase is a ubiquitous and multifunctional enzyme that can catalyze hydroxylation and successive oxidation of phenolic compounds to quinones, regulating the melanogenesis process in humans. 

Moreover, according to a preliminary *in vitro* study, *L. shawii* extracts have been reported to show antimicrobial effects on different species of bacteria [23]. Indeed, the anti-*Candida* activity of plants rich in polyphenols and polymeric flavan-3-ols has been studied [24,25,26]. Among the monoterpene phenols, carvacrol was investigated for its antifungal and antibacterial effects [27], and different studies demonstrated that this molecule not only is able to explicate a potent action against *C. albicans*, but also impairs the growth of different morphological forms, such as yeast, hyphae, and even the most resistant forms of biofilm [28].

Considering the multifactorial parameters, such as origin, cultivar, harvest dates, climatic factors, and applied technologies that could severely impact on the phytochemical profile and consequently on the associated biological activity, the objective of the present work is to determine the content and composition of the main functional constituents of four goji berry cultivars harvested in Italy and Romania at different dates with a particular emphasis on the polyphenolic derivatives. Their antioxidant potential, inhibitory activity on tyrosinase, and anti-fungal activity after two different treatments of homogenization (Ultraturrax^®^ IKA, Staufen, Germany, or domestic mixer Girmi, Omegna, Italy) were evaluated. Besides the total phenolic and flavonoid content, the antioxidant capacity was evaluated using three assays—2,2-diphenyl-1-picryl-hydrazyl (DPPH), 2,2′-azino-bis-(3-ethylbenzothiazoline-6-sulfonate (ABTS), and ferric reducing antioxidant potential (FRAP)—to detect the radical scavenging activity of the investigated samples.

## 2. Materials and Methods

### 2.1. Materials

All HPLC-grade solvents were purchased from Carlo Erba (Milan, Italy) and Fluka (Milan, Italy), whereas chemical standards were supplied by Sigma-Aldrich (Milan, Italy).

Goji berries were generously gifted by Azienda Natural Goji^®^ (Fondi, Italy), and by two local providers from Northwest Romania. Samples given by Azienda Natural Goji^®^, belonging to “Poland” and “Wild” varieties, P and W, were harvested at different commercial harvesting periods (2015: 6 July, 23 July, 3 August; 2016: 26 July; 4 August, P1–P5 and W1–W5) in Fondi (Italy) based on their stage maturity as determined by the producer. Samples given by the two local providers from Romania belonged to the Biglifeberry and Erma (B, E) varieties, were from only one collection (B1, E1), and were harvested at maturity in the summer of 2014 from two organic cultures from Ciuperceni (E variety, SM county) and Ploscoș (B variety, Cluj county). No specific permissions were requested for the plant sample collection, which did not involve endargered or protected species.

All samples were quickly frozen at −80 °C and stored at −18 °C until the analyses were performed, with no direct sunlight exposure.

### 2.2. Sample Preparation

Sample preparation was made according to the method previously described [10]. In brief, defrosted, washed, and wiped goji berries were homogenized at room temperature for 2 min by a T18 Ultraturrax^®^ homogenizer (IKA^®^, Staufen, Germany) at 10,000 rpm (U samples) or by a domestic mixer at 16,000 rpm (TR01, Girmi, Omegna Italy) (D samples), and the resulting purees were subjected to the extraction procedure. Aliquots of fresh whole and homogenized samples were dehydrated to constant weight with the only aim to evaluate their water content.

### 2.3. Extraction of Hydroalcoholic Fractions

Extractions were performed with a hydroalcoholic mixture (ethanol:water = 70:30, water acidified with 0.5% acetic acid) and a small amount of cyclohexane for 3 h. The resulting suspension was centrifuged at 12,000× *g* for 10 min at 4 °C, and the upper organic phase was discarded. The hydroalcoholic phase, whose separation from the lower solid phase was completed using paper filtration, was transferred to a rotary evaporator at a reduced pressure and 40 °C. The dryness was completed using lyophilization. The obtained residues, protected from light and air exposure, were either immediately analyzed or stored at –18 °C until further analyses.

### 2.4. HPLC Analysis

Chromatographic analyses were carried out following a validated method [29] for the detection and quantification of the main secondary metabolites.

Moreover, rutin was quantified at 360 nm according to a slightly modified method based on the method reported by Imbaraj et al. [30] and described in a previous work [10]. Rutin was identified via comparison of the retention time and of the UV spectra of a pure external standard and quantified using a calibration curve (*y* = 62*x* – 0.02, *R*^2^ = 0.9972, in the range 0.5–50 µg/mL). Quercetin and kaempferol derivatives were approximately identified using the evaluation of the UV spectra and indicatively quantified as rutin equivalents.

### 2.5. Total Phenolic Content (TPC)

The TPC was assessed using the Folin-Ciocâlteu method [21] with a SPECTROstar Nano Multi-Detection Microplate Reader in 96-well plates (BMG Labtech, Ortenberg, Germany). Briefly, a mixture solution consisting of 20 µL of extract, 100 µL of Folin-Ciocâlteu reagent, and 80 µL of sodium carbonate (Na_2_CO_3_, 7.5% *w/v*) was homogenized and incubated at room temperature in the dark (30 min). The TPC was expressed as gallic acid equivalents (GAE) in milligrams per gram dry weight (dw) of plant material (mg GAE/g dw plant material) after reading the absorbance of the samples at 760 nm. 

### 2.6. Total Flavonoid Content (TFC)

The total flavonoid content (TFC) was determined using the AlCl_3_ method, with the absorbance of the samples being measured at 420 nm. The results of the TFC were expressed as quercetin equivalents (QE) in milligrams per gram dry weight (dw) of plant material (mg QE/g dw plant material) [31].

### 2.7. Antioxidant Activity

#### 2.7.1. DPPH Radical Scavenging Assay

The DPPH antioxidant capacity was determined according to the method described in Mocan et al. [32], with some modifications, by using a SPECTROstar Nano microplate reader (BMG Labtech, Offenburg, Germany). Each of the 96 wells consisted of 30 µL of sample solution (in an appropriated dilution) and a 0.004% methanolic solution of DPPH. After 30 min of incubation in the dark, the absorbance of the sample was read at 515 nm. Results were expressed as Trolox equivalents per gram of dry weight herbal extract (mg TE/g dw herbal extract).

#### 2.7.2. Trolox Equivalent Antioxidant Capacity (TEAC) Assay

The TEAC of the samples was measured using the method previously described [21] and the results were expressed as milligrams of Trolox equivalents (TE) per gram of dry herbal extract (mg TE/g dw extract).

#### 2.7.3. FRAP Assay

The FRAP assay was assessed using the method described in Damiano et al. [33] with slight modifications. The FRAP reagent was prepared by mixing ten volumes of acetate buffer (300 mM, pH 3.6), one volume of 2,4,6-tris(2-pyridyl)-*s*-triazine (TPTZ) solution (10 mM TPTZ in 40 mM HCl), and one volume of FeCl_3_ solution (20 mM FeCl_3_·6H_2_O in 40 mM HCl). After mixing the samples with the reagent (25 µL sample and 175 µL FRAP reagent), the samples were incubated in the dark for 30 min at room temperature and the absorbance was measured at 593 nm in 96-well plates. The final results were expressed as milligrams of Trolox equivalents (TE) per milligram of extract (mg TE/g extract).

### 2.8. Tyrosinase Inhibitory Activity

The tyrosinase inhibitory activity of each sample was determined via a method previously described [34] using a SPECTROstar Nano Multi-Detection Microplate Reader with 96-well plates (BMG Labtech, Ortenberg, Germany). Samples were disolved in water containing 5% dimethylsulfoxide (DMSO). For each sample, four wells were designated as A, B, C, and D, where each one contained a reaction mixture (200 µL) as follows: (A) 140 µL of 66 mM phosphate buffer solution (pH = 6.6) (PBS), 40 µL of mushroom tyrosinase in PBS (23 U/mL) (Tyr); (B) 160 µL PBS; (C) 80 µL PBS, 40 µL Tyr, 40 µL sample, and 80 µL PBS; and (D) 120 µL PBS and 40 µL sample. The plate was then incubated at room temperature for 10 min; after incubation, 40 µL of 2.5 mM L-DOPA in PBS solution were added in each well and the mixtures were incubated again at room temperature for 20 min. The absorbance of each well was measured at 475 nm and the inhibition percentage of the tyrosinase activity was calculated using the following Equation (1):

(1) $$\%I=\frac{\left(A\;–B\right)–\left(C-D\right)}{\left(A\;–B\right)}\text{×}100$$

The results were expressed as mg kojic acid equivalents per gram of dry weight of extract (mg KAE/g extract) using a calibration curve between 0.01–0.10 mg kojic acid/mL of solution.

### 2.9. Antifungal Susceptibility Testing

The *in vitro* antifungal susceptibility was evaluated using extracts, rutin, and carvacrol (Sigma Aldrich, St. Louis, MO, USA). To evaluate the minimal inhibitory concentration (MIC) of the extracts and compounds, a broth microdilution method was performed according to a standardized protocol for yeasts [35].

The assay was carried out with *C. albicans* ATCC 24433, *C. albicans* ATCC 10261, and *C. albicans* ATCC 90028 coming from the American Type Culture Collection (ATCC, Rockville, MD, USA). *Candida* strains were grown on Sabouraud dextrose agar at 37 °C for 24 h. Then, cell suspensions of the strains were prepared in a RPMI 1640 medium (Sigma-Aldrich, Milan, Italy) buffered to pH 7.0 with 0.165 mmol L^–1^ 3-(*N*-morpholino)propanesulfonic acid (MOPS). The final concentration of the inoculum was 1 × 10^3^–5 × 10^3^ cells mL^–1^. The extracts were dissolved in DMSO and diluted 100 times in RPMI 1640 broth. Ten concentrations ranging from 1000 to 1.9 μg mL^–1^ for the extracts and from 64 to 0.125 μg mL^–1^ for the compounds were tested against *Candida albicans* strains in 96-well round-bottom microtitration plates. The MIC_50_, MIC_90_, and MIC_100_—the lowest concentrations of extracts that caused growth inhibitions ≥50%, ≥90%, and 100%, respectively—were evaluated. Data was reported as the median of the MIC.

## 3. Results and Discussion

### 3.1. Polyphenols Extraction and HPLC Analysis

The selected freshly defrozen berry samples were submitted to a mild and quick, one-step double-phase extraction (graphical abstract), which allowed us to obtain the hydroalcoholic fraction separated by the solid residue and purified by the lipid fraction as already described in a previous work [10]. The only centrifugation step, followed by a preliminary evaporation of ethanol and the subsequent freeze-drying, allowed for obtaining a residue that was directly ready for the HPLC analysis and for the evaluation using the biological tests.

Fresh samples as such, or after homogenization, were also submitted to a dehydration step to evaluate their water content (about 88%), and the dried samples were extracted in the same conditions that were applied to the fresh samples. Results, not shown, demonstrate that the dehydration process did not influence the extraction yield nor the chemical composition of the hydroalcoholic fraction.

Extraction yields (% *w/w*) of the hydroalcoholic fraction from goji berries performed with the above-described conditions from the selected samples accounted for 35–60% in dry weight with mean values around 43–46%. No statistically relevant differences were observed comparing cultivars type, harvesting date, seasons, or adopted homogenization technique. Indeed, the homogenization process, as shown later, had an influence on the quali-quantitative distribution of each analyte. A lower yield (medium 378 vs 523 mg/g dry weight) was shown for the harvesting dates of season 2016 with respect to those of 2015, but only for sample P. This could depend on the different maturation stages of the selected samples, but it seems not relevant for an overall rating.

### 3.2. HPLC Analysis at 278 nm

From the analysis of the data reported in Table 1 regarding the phenolic profiling of goji berries, we can first note that, among the 21 phenolic standards, vanillic acid, 4-hydroxy benzoic acid, 3-hydroxy-4-methoxy benzaldehyde, *p*-coumaric acid, *t*-ferulic acid, naringin, 2,3-dimethoxy benzoic acid, benzoic acid, *o*-coumaric acid, quercetin, harpagoside, and *t*-cinnamic acid were not detected or were present in trace amounts (below Limit Of Quantification or Limit Of Detection, data not shown). The inter- and intra-cultivar differences were huge and did not follow a specific trend. Generally speaking, Italian cultivars were richer in terms of phenolic compounds with respect to Romanian ones. Among Italian cultivars (especially for Wild), the last two harvesting dates gave berries richer in phenolic compounds regardless of the mixing treatment. Chlorogenic acid, carvacrol, and at lower extent, sinapinic acid were the most-detected metabolites. Conversely, gallic acid, catechin, and syringic acid were only present in some cultivars and their amounts differed a lot depending on the applied treatment. Lastly, a few samples were also characterized by low amounts of naringenin (P2D) and epicatechin (P4U).

The chromatograms of the flavonoid fraction, recorded at 360 nm, showed the presence of about 10–15 peaks, according to the different analyzed cultivars, among which the most representative was rutin. In Table 2, rutin and the total flavonoid contents are reported, calculated as rutin equivalents, after considering all the peaks whose areas were at least 10% of rutin area and taking into account the standard calibration curve of this molecule. Values are expressed in relation to dry weight (dw) for a comparison with the literature data and in μg/mg extract for a better evaluation of other data on bioactivity presented in this work and obtained by the extracts evaluation. Rutin accounted for about 185–400 µg/g dw of goji berries. In the literature, reported values of 281 [30], 296 [36], and 326 µg/g dw [37] are found, perfectly overlapping with our results. Wider ranges between 159–629 µg/g dw were reported in Zhang et al. [38] and higher values (730–1380 µg/g dw) were detected in Dong et al. [39]. Many different results were available in the literature regarding the other representative flavonoids recognized mainly as quercetin hexosides, and secondly as kaempferol and isorhamnetin derivatives. Zhang et al. [38] reported the quercetin-rhamno-di-hexoside content as 435–1065 µg/g, Donno et al. [14] reported a hyperoside content of 116 mg/100 g fresh weight, whereas Inbaraj et al. [30] studied different quercetin-rhamno-hexoside derivatives (in the range of 70–440 µg/g). In our chromatograms, many different peaks were detected in the flavonoid component, although their identification was not possible. These were reported as a sum and expressed as rutin equivalents on the basis of its calibration curve. According to this evaluation, rutin accounted for about 20–65% of the total flavonoid components. The selected samples did not show relevant differences among cultivars (rutin content mean values: P = 276 ± 8.3, W = 291 ± 8.7, E = 288 ± 8.6, and B = 255 ± 7.6 µg/g), nor among applied homogenization processes (D = 287 ± 8.6 vs U = 279 ± 8.4 µg/g), whereas differences could be revealed in the rutin content along the maturation stage (i.e., rutin increased by 70%, from P1 to P3, collected a month apart in July–August 2015 and decreased by 45% from P4 to P5, collected fifteen days apart, in July–August 2016). The total flavonoid content ranged from 380–1600 µg/g with a slight difference between cultivars P and W (770 ± 38 vs 910 ± 45 µg/g) and no differences after homogenization treatments D and U (815 ± 41 vs 862 ± 43 µg/g).

As shown by the data, the rutin content increase was usually correlated with a decrease in the amount of other flavonoid components and vice versa, but this trend was not confirmed by the P4–P5 series. The comparison between the D and U series demonstrated differences between P4D and P4U samples only in terms of an increase of other flavonoids, whereas W5D and W5U samples were characterized by discrepancies in terms of rutin and flavonoid amount. Overall, the different ratios between rutin and other flavonoids in the goji phytocomplex depended on the cultivar type, but cultivars also seemed highly influenced by the maturation step, as is clearly shown by the sample P.

### 3.3. Total Phenolic and Flavonoid Content

The total phenolic (TPC)/flavonoid (TFC) content assays are rapid and low-cost methods used for the screening of herbal or food samples subjected to different processing or treatment procedures [39,40]. In this study, the highest value for the TPC was found in the W4U sample, followed by P4D, whereas the highest TFC was obtained from the P4U and W4U samples, as seen in Table 3. Interestingly, the lowest values for the TPC were presented by the samples collected from Romania, while Italian samples presented overall higher values for TPC. The results obtained in this study for the Italian samples are comparable with the ones obtained for samples collected in the Umbria region (central Italy) [41], while the results obtained for the Romanian samples are more comparable with the findings concerning samples collected from Northern Italy [14]. Furthermore, these results support the evidence that TPC in our tested goji berries could vary among different genotypes and was strictly dependent on the pre-harvest practices, cultivar/clone, environmental conditions, and maturity stage at harvest. All these experimental variables could modulate the accumulation of specific phenolics [42], which are majorly responsible of the antioxidant and biological activity.

### 3.4. Antioxidant Activity

The antioxidant capacity of plant extracts and food matrices can be evaluated using several *in vitro* assays. Since the results of the antioxidant effect generated by an assay are influenced by the used protocol, a combination panel of assays is being required. The antioxidant capacity of the several goji berries cultivars was tested using three different assays to underline the different antioxidant facets of the investigated samples (Table 3).

#### 3.4.1. Trolox Equivalent Antioxidant Capacity (TEAC) Assay

The TEAC assay measured the ability of goji berries’ phenolic extracts to reduce the *in vitro* formed radicals. In this study, among Italian samples, the highest TEAC value was presented by sample W4U (63.58 ± 13.07 mg TE/g extract), which also presented high values for TPC and TFC. However, the highest value for the TEAC assay was obtained from the Romanian B1D sample (76.78 ± 7.91 mg TE/g extract). Zhang et al. [38] reported TEAC values ranging from 47.8 ± 6.6 to 78.2 ± 4.8 µM TE/g fresh weight for eight different Chinese goji berries genotypes, while Abdennacer et al. [43] reported a TEAC value of 0.08 mg TE/mg dw for *Lycium intricatum* Boiss. fruit methanolic extracts. Nonetheless, Mocan et al. [15] obtained values of 24.86 ± 2.15 and 25.12 ± 2.11 mg TE/g extract for 70% methanolic extracts obtained using sonication of the same Romanian cultivars of goji berries.

#### 3.4.2. DPPH Radical Scavenging Assay

The DPPH radical scavenging assay is among the most frequently used antioxidant assays and offers a first approach for evaluating the antioxidant capacity of a complex mixture [44]. In the current research, the DPPH activity of the tested goji berries ranged from na (not active, B1D sample) to the highest value of 40.32 ± 4.01 mg TE/g extract (for the Italian W5D sample). Zhang et al. [38] obtained values ranging between 35.88 ± 2.2 to 85.46 ± 1.9 µM TE/g fresh weight for the DPPH antioxidant capacity assay from eight Chinese goji genotypes, while Abdennacer et al. [43] reported a value of 0.05 mg TE/mg dry weight for the methanolic extracts of fruits of Tunisian *Lycium intricatum*.

#### 3.4.3. FRAP Assay

The FRAP assay is an electron transfer-based method that measures the reduction of the ferric ion (Fe^3+^)-ligand complex to the intensely blue colored ferrous (Fe^2+^) complex via antioxidants in an acidic environment [44]. In this study, the highest reducing power was presented by the Italian W4U sample with a value of 51.93 ± 2.97 mg TE/g extract, followed by the P4D sample (36.63 ± 0.84 mg TE/g extract), while the lowest value was obtained for Italian W2U sample (15.71 ± 0.05 mg TE/g extract).

Overall, the different trends and results obtained in the antioxidant capacity assays show that the contents of total phenolics and flavonoids could not sufficiently explain the observed antioxidant activity of fruit and plant phenolic extracts, which are mixtures of different compounds with various activities in the tested samples [42].

### 3.5. Enzyme Inhibitory Activity

Tyrosinase inhibitors can be attractive for cosmetic and pharmaceutical industries as depigmentation agents, as well as in food and agriculture industries as anti-browning compounds. Goji berry extracts have been previously described as tyrosinase inhibitors [15] and are currently used as natural ingredients in several market-available cosmetic or dermato-cosmetic preparations. In this study, the tyrosinase inhibitory activity of the tested goji extracts ranged between 1.56 ± 0.74 mg KAE/g extract for the W3U sample to 62.79 ± 0.66 for the W4U sample. The second-highest value in terms of tyrosinase inhibition was obtained for the Romanian E1D sample (59.49 ± 1.33 mg KAE/g extract), while the two samples from Romania generally exhibited a higher tyrosinase inhibitory potential than samples from Italy, except sample W4U.

### 3.6. Anti-Candida Activity of Extracts

Antifungal activity was demonstrated via a broth microdilution method, using the standard drug fluconazole as a positive control. Among the different goji extracts tested against three *Candida albicans* strains, P samples displayed the best inhibitory activity. In particular, P1D, P1U, W1D, W1U, E1D, and B1D showed MIC_50_ values of 138 µg mL^–1^, 186 µg mL^–1^, 238 µg mL^–1^, 250 µg mL^–1^, >1000 µg mL^–1^, and >1000 µg mL^–1^, respectively, against all *Candida albicans* strains. Samples harvested at different commercial harvesting periods showed different MIC values. Interestingly, the P1D sample, harvested on 6 July 2015, was endowed with the best antifungal activity compared to the other harvesting periods (P2D, P3D, P4D and P5D) with a MIC_100_ of 476 µg mL^–1^, 552 µg mL^–1^, 609 µg mL^–1^, >1000 µg mL^–1^, and >1000 µg mL^–1^, respectively against all the considered strains (Table 4).

Due to the presence in these extracts of considerable amounts of well-recognized antimicrobial metabolites, we further tested rutin and carvacrol against the same fungal strains [45]. Rutin was almost inactive with MIC values ≥64 μg/mL. Conversely, carvacrol displayed a strong inhibitory activity against *C. albicans* with MIC_50_ ranging from 0.125 μg/mL to 0.25 μg/mL. Despite the high amount of carvacrol present in B1D (Table 1), it displayed a weak anti-fungal activity thus suggesting that the concurrent presence of other secondary metabolites can enhance or decrease the bioactivity of these goji extracts.

Furthermore, considering the quantified components and the explicated anti-*Candida* effects, W4U represented the sample characterized by a higher amount of detected secondary metabolites and a positive upward trend in the biological activity. Many components, other than those quantified using the HPLC analyses, could contribute to the TPC and TFC of the W4U sample, whose activity was evidently correlated not only with the single analytes, even though they represented the most representative molecules in weight terms, but also in the complexity of the phytocomplex, evidently characterized by many different minor components. However, this clearly shows how important the work-up is in the preservation of many components (especially for flavonoid compounds, considering the effect of U on P) represented in extremely low concentrations. The high flavonoid component seemed to correlate, in particular, with a marked activity on tyrosinase.

## 4. Conclusions

Despite the long tradition in nutrition and medicine, the goji berry’s composition is strictly related to the geographical origin (soil type, climate), cultivar type, harvesting time, and industrial/domestic processing. The changes of the phytochemical profile have a deep influence on the bioactivity of this herbal medicinal/edible product. Our investigation aimed at the identification of the main bioactive components that are responsible for selected biological activities. The HPLC analytical fingerprint was characterized by a variable amount of phenolic compounds, mainly depending on the geographical origin, cultivar type, homogenization treatment, and above all, harvesting period. Moreover, we highlighted, by means of an array of different assays, this valuable natural matrix in terms of the antioxidant activity, anti-fungal activity, and inhibition of tyrosinase, aiming at the correlation between the phytochemical profile and the reported bioactivity. These data could be of high interest in the formulation of new goji-based food supplements and for further pharmaceutical development of this valuable plant matrix.

## Figures and Tables

**Table 1:** Phenolic pattern of goji berries.

Compound	Concentration (μg/mg ± SD)																					
	P1D	P2D	P3D	P4D	P5D	P1U	P2U	P3U	P4U	P5U	W1D	W2D	W3D	W4D	W5D	W1U	W1U	W3U	W4U	W5U	E1D	B1D
Gallic acid		0.77 ± 0.07				0.03 ± 0.01		tr														
Catechin						0.23 ± 0.01			0.23 ± 0.01		tr				0.37 ± 0.01		0.03 ± 0.01		0.31 ± 0.01	0.25 ± 0.01		
Chlorogenic acid		0.19 ± 0.01	0.16 ± 0.01	0.55 ± 0.02	0.54 ± 0.02	0.56 ± 0.01	0.11 ± 0.01	0.13 ± 0.01	0.57 ± 0.02	0.37 ± 0.02	0.47 ± 0.02	0.10 ± 0.01		0.77 ± 0.03	0.43 ± 0.02	0.22 ± 0.01	0.12 ± 0.01	0.28 ± 0.01	0.43 ± 0.02	0.45 ± 0.02		
Epicatechin									0.07 ± 0.01													
Syringic acid										0.06 ± 0.01			0.14 ± 0.01	0.09 ± 0.01			0.04 ± 0.01	0.21 ± 0.01	0.27 ± 0.01			
3-Hydroxy benzoic acid															0.08 ± 0.01							
Sinapinic acid			tr	0.03 ± 0.01	0.03 ± 0.01	0.03 ± 0.01		tr	0.03 ± 0.01	0.03 ± 0.01	tr			0.03 ± 0.01	0.03 ± 0.01	tr			0.03 ± 0.01	0.03 ± 0.01		
Naringenin		0.06 ± 0.01																				
Carvacrol	0.03 ± 0.01	0.05 ± 0.01	0.05 ± 0.01	0.05 ± 0.01	0.05 ± 0.01	0.08 ± 0.01	0.05 ± 0.01	0.04 ± 0.01	0.05 ± 0.01	0.04 ± 0.01	0.04 ± 0.01	0.06 ± 0.01	0.05 ± 0.01	0.04 ± 0.01	0.07 ± 0.01	0.04 ± 0.01	0.05 ± 0.01	0.05 ± 0.01	0.04 ± 0.01	0.05 ± 0.01		0.23 ± 0.02
**Total (μg/mg)**	0.03	1.07	0.21	0.63	0.62	0.93	0.16	0.17	0.95	0.50	0.51	0.16	0.18	0.93	0.98	0.27	0.24	0.54	1.09	0.78		0.23

tr: traces; P: Polonia cultivar; W: Wild cultivar; E: Erma cultivar; B: Biglifeberry cultivar; D: domestic mixer; U: Ultraturrax^®^.

**Table 2:** Rutin and flavonoid content of analyzed goji berries hydroalcoholic (HA) extracts. Flavonoids are expressed as rutin equivalent using the HPLC areas collected at 360 nm.

Sample	HA Extract (mg/g dw)	Rutin (± 3%) (µg/g dw)	Flavonoids (± 5%) (µg/g dw)	Rutin (± 3%) (µg/mg Extract)	Flavonoids (± 5%) (µg/mg Extract)
P1D	467	210	790	0.45	1.69
P2D	548	263	661	0.48	1.28
P3D	528	360	658	0.68	1.25
P4D	410	330	950	0.80	2.31
P5D	340	205	564	0.60	1.66
W1D	422	371	995	0.88	2.36
W2D	472	274	699	0.58	1.48
W3D	493	256	874	0.52	1.77
W4D	430	268	1033	0.62	2.40
W5D	448	336	925	0.75	2.06
P1U	450	184	591	0.41	1.31
P2U	558	335	735	0.60	1.32
P3U	589	316	705	0.54	1.20
P4U	414	317	1598	0.77	3.86
P5U	345	238	428	0.69	1.24
W1U	456	405	1134	0.89	2.49
W2U	506	309	642	0.61	1.27
W3U	494	242	1237	0.49	2.50
W4U	464	198	1183	0.43	2.55
W5U	413	250	374	0.60	0.90
E1D	528	288	1605	0.54	3.04
B1D	420	255	752	0.61	1.79

dw: dry weight.

**Table 3:** Total phenolic (TPC) and flavonoid (TFC) content, 2,2-diphenyl-1-picryl-hydrazyl (DPPH), 2,2′-azino-bis-(3-ethylbenzothiazoline-6-sulfonate (ABTS) scavenging capacity, ferric reducing antioxidant power (FRAP), and tyrosinase inhibition of the extracts of *Lycium* cultivars (values expressed are means ± SD of three measurements).

Sample	TPC (mg GAE/g dw Extract)	TFC (mg QE/g dw Extract)	FRAP (mg TE/g dw Extract)	DPPH Scavenging (mg TE/g dw Extract)	ABTS Scavenging (mg TE/g dw Extract)	Tyrosinase Inhibition (mg KAE/g dw Extract)
P1D	19.22 ± 0.52	4.05 ± 0.61	23.84 ± 0.31	26.29 ± 1.65	40.45 ± 0.26	2.12 ± 0.25
P2D	17.09 ± 1.59	3.58 ± 0.11	19.47 ± 0.99	24.62 ± 0.24	34.79 ± 1.13	2.50 ± 0.86
P3D	18.09 ± 0.13	3.87 ± 0.76	20.80 ± 0.74	16.77 ± 4.38	36.85 ± 0.39	2.39 ± 0.53
P4D	22.64 ± 0.97	2.89 ± 0.13	36.63 ± 0.84	36.47 ± 1.42	44.97 ± 0.48	9.69 ± 0.19
P5D	19.50 ± 0.67	2.64 ± 0.17	33.28 ± 2.03	35.58 ± 2.14	44.91 ± 0.61	6.54 ± 0.78
W1D	12.28 ± 1.87	3.18 ± 0.21	16.76 ± 1.03	18.66 ± 2.22	31.88 ± 0.58	2.59 ± 0.35
W2D	20.29 ± 0.88	4.26 ± 0.13	21.24 ± 1.46	16.77 ± 2.83	39.10 ± 1.68	3.17 ± 0.83
W3D	15.33 ± 0.16	3.45 ± 0.13	20.96 ± 0.75	17.99 ± 3.98	37.71 ± 0.33	3.33 ± 0.70
W4D	15.27 ± 0.69	2.39 ± 0.06	29.90 ± 1.57	32.97 ± 2.60	37.84 ± 0.74	8.03 ± 0.85
W5D	19.54 ± 0.70	3.56 ± 0.24	32.14 ± 3.49	40.32 ± 4.01	42.03 ± 0.20	8.42 ± 0.75
P1U	18.86 ± 0.45	3.42 ± 0.10	28.54 ± 0.59	32.58 ± 2.70	40.67 ± 1.16	5.05 ± 0.58
P2U	18.39 ± 1.57	3.87 ± 0.26	25.93 ± 1.79	21.78 ± 3.53	39.10 ± 0.46	3.50 ± 0.29
P3U	19.59 ± 0.73	3.61 ± 0.14	23.86 ± 0.59	21.11 ± 3.94	38.02 ± 0.63	3.72 ± 0.63
P4U	7.69 ± 1.78	11.03 ± 1.24	23.89 ± 2.09	10.75 ± 0.06	45.24 ± 4.69	57.60 ± 0.01
P5U	17.71 ± 0.93	2.77 ± 0.17	30.86 ± 0.86	26.23 ± 2.15	39.43 ± 0.46	6.48 ± 0.16
W1U	17.43 ± 0.30	4.02 ± 0.02	20.90 ± 0.49	19.89 ± 1.65	37.57 ± 1.13	4.33 ± 0.72
W2U	11.83 ± 0.67	3.13 ± 0.24	15.71 ± 0.05	12.20 ± 2.53	27.81 ± 0.46	4.66 ± 0.99
W3U	17.00 ± 0.65	3.24 ± 0.12	20.49 ± 0.62	17.99 ± 0.77	35.96 ± 0.38	1.56 ± 0.74
W4U	23.49 ± 3.36	10.54 ± 0.12	51.93 ± 2.97	14.98 ± 4.40	63.58 ± 13.07	62.79 ± 0.66
W5U	15.10 ± 1.41	2.58 ± 0.09	28.59 ± 2.55	29.13 ± 0.94	35.76 ± 0.30	9.30 ± 0.44
E1D	5.76 ± 0.24	10.47 ± 1.83	23.00 ± 3.66	2.18 ± 0.84	51.14 ± 4.27	59.49 ± 1.33
B1D	3.31 ± 0.66	9.29 ± 0.60	21.56 ± 2.28	na	76.78 ± 7.91	49.35 ± 4.33

na: not active.

**Table 4:** Antifungal activity of goji berry extracts and reference compounds expressed as an MIC (µg/mL).

Sample	MIC (μg/mL)* at 24 h								
	*Candida albicans* ATCC 24433			*Candida albicans* ATCC 10261			*Candida albicans* ATCC 90028		
	50	90	100	50	90	100	50	90	100
P1D	125	250	500	281.25	312.5	625	93.75	250	500
P2D	250	250	500	375	375	750	125	250	500
P3D	250	250	500	375	500	1000	187.5	250	500
P4D	125	1000	>1000	500	1000	>1000	500	1000	>1000
P5D	250	1000	>1000	500	1000	>1000	500	1000	>1000
P1U	125	187.5	375	375	500	1000	250	375	750
P2U	250	250	500	375	500	1000	250	250	500
P3U	250	250	500	375	500	1000	250	250	500
P4U	125	250	1000	250	500	1000	250	500	1000
P5U	250	500	1000	250	500	1000	500	1000	1000
W1D	250	250	500	500	500	1000	187.5	250	500
W2D	250	250	500	500	500	1000	187.5	250	500
W3D	250	250	500	500	500	1000	250	250	500
W4D	125	1000	>1000	500	1000	>1000	500	1000	>1000
W5D	125	1000	>1000	500	1000	>1000	500	1000	>1000
W1U	187.5	250	500	500	500	1000	250	375	500
W2U	250	375	500	500	500	1000	375	375	750
W3U	250	250	500	500	500	1000	125	250	500
W4U	250	250	>1000	500	1000	>1000	500	1000	>1000
W5U	250	1000	>1000	500	1000	>1000	500	1000	>1000
E1D	>1000	>1000	>1000	>1000	>1000	>1000	>1000	>1000	>1000
B1D	>1000	>1000	>1000	>1000	>1000	>1000	>1000	>1000	>1000
Rutin	64	>64	>64	64	>64	>64	64	>64	>64
Carvacrol	0.25	1	2	0.25	1	2	0.125	0.25	0.5
Fluconazole	0.5	2	8	1	4	>64	0.5	2	8

* The data are expressed as a median.
